# Acquired retinoschisis resolved after 23Gage pars plana vitrectomy in posterior microphthalmos

**DOI:** 10.1186/1471-2415-14-65

**Published:** 2014-05-11

**Authors:** Shanshan Yu, Yi Gao, Xiaoling Liang, Yongsheng Huang

**Affiliations:** 1 State Key Laboratory of Ophthalmology, Zhongshan Ophthalmic Center, Sun Yat-Sen University, 54, S. Xianlie Road, Guangzhou 510060, P.R. China

**Keywords:** Microphthalmos, Acquired retinoschisis, Pars plana vitrectomy

## Abstract

**Background:**

Posterior microphthalmos combined with acquired retinoschisis is a rare entity. This report presents a case of acquired retinoschisis in a patient with posterior microphthalmos and discusses the management for such disease. The patient exhibited acquired peripheral retinal schisis in both eyes.

**Case presentation:**

The patient presented with a fix scotoma and decrease in visual acuity for 2 weeks in his left eye. Ocular examination revealed that his best-corrected visual acuity was 0.6 in right eye and 0.2 in left eye. The patient had amblyopia because of hyperopia with spherical equivalent of +11.75 diopters in the right eye and +12.00 diopters in the left eye. The axial lengths were 18.41 mm in right and 18.43 mm in left eyes respectively. Slip lamp examination found normal anterior segments. Funduscopy showed bilateral retinoschisis in inferotemporal retina. The schisis in right eye was limited to peripheral retina whereas the schisis in left eye was bullous type. The schisis in the left eye extended from the periphery to the posterior macular region in left eye. A pars plana vitrectomy was performed in the left eye and visual acuity was restored to 0.6.

**Conclusion:**

Posterior microphthalmos combined with retinoschisis is rare. When it appears in peripheral retina, the schisis remains stable. In cases where the schisis extends to posterior pole and affects the macula, surgery in the form of pars plana vitrectomy could be an option.

## Background

Posterior microphthalmos (PM) is a developmental defect in which affected eyes display vitreous chamber foreshortening, and papillomacular retinal folds with normal or nearly normal anterior chamber depth, [1]. PM is a relatively rare. It usually coexists with high hyperopia, glaucoma, uveal effusion syndrome, and exudative retinal detachment [2,3]. PM combined with foveoschisis has been reported in some cases [4,5] and has been related to gene mutation [4,6]. Prevously, schisis has been reported in 3 cases with nanophthalmos [7], in which the schisis was limited to the peripheral retina. We present a case with PM and bullous-like retinoschisis in a hyperopic eye.

## Case presentation

**Figure 1:**
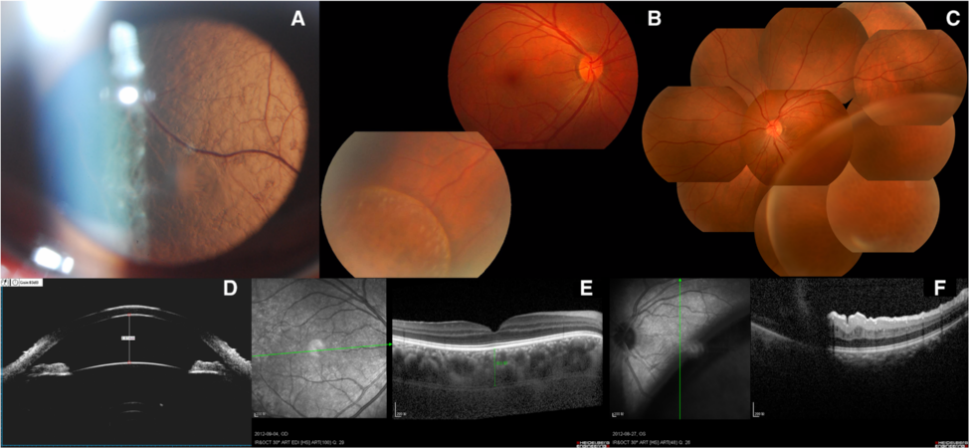
**Baseline statue of the patient. A**: ASP OS: smooth thinner schisis retina nearly contact to the len. **B**: FP OD: Retinoschisis limited in inferotemporal peripheral retina of right eye. **C**: FP OS: Retinoschisis presented a bullous, smooth, thinner surface in left eye. **D**: UBM OS: showed normal anterior camber. **E**: OCT OD: normal fundus with a thick neurosensory retina and a thick choroid (503 μm). **F**: OCT OS: macular folds caused by the elevated schisis compression. ASP: anterior segment photography; FP: fundus photography; UBM: ultrasound biomicroscopy; OCT: Optical coherence tomography.

A 35-year-old Chinese man complained of a fix scotoma and decrease in visual acuity for 2 weeks in his left eye. He received amblyopia therapy when he was 4, because of amblyopia related to high hyperopia. The best-corrected visual acuity (BCVA) was 0.6 in his right eye (OD) and 0.2 in the left eye (OS) (spherical equivalent +11.75D OD, +12.00D OS). The corneal diameter was 10.5 mm in both eyes (OU), anterior chamber depths were 2.87 mm OD and 2.81 mm OS (Figure 1D), and, total axial lengths were 18.41 mm OD and 18.43 mm OS, respectively. A diagnosis of posterior microphthalmos was made. The morphology of schisis in two eyes was different. The schisis was limited in right eye (Figure 1B) whereas it presented as bullous, smooth elevation with thin surface in the left eye (Figure 1C). The bulla in left eye was touching the posterior surface of the lens (Figure 1A) and compressed the macular region resulting in macular folds and edema. Ultrasound biomicroscopy revealed peripheral cystic degeneration OU, without ciliary body or choroidal detachment. Fundus fluorescence angiography (FFA) showed peripheral vessels leakage OU, without background fluorescence changes.

Spectralis optical coherence tomography (OCT) scan showed a thick retinaal neurosensory layer and a thick choroid (503 μm) in right eye (Figure 1F). In left eye, the schisis was too high to obtain an image (Figure 1G).

In order to exclude retinoschisis related to possible inflammation, a short-term course of systemic corticosteroids was used. Methylprednisolone was started at a dose of 1.6 mg/kg/day for 3 days, followed by 0.8 mg/kg/day for 3 days and 0.4 mg/kg/day for 5 days. However, the bulla showed no change. Next, we performed 23 G pars plana vitrectomy in the left eye. Before surgery, a careful scleral depression examination was performed to rule out any outer or inner layer breaks of retina. Triamcinolone acetonide associated posterior vitreous detachment was performed. After an internal drainage hole was made, the bulla collapsed. This was followed by photocoagulation in schisis region under perfluorocarbon liquid. This was followed by tamponade with C3F8.

**Figure 2:**
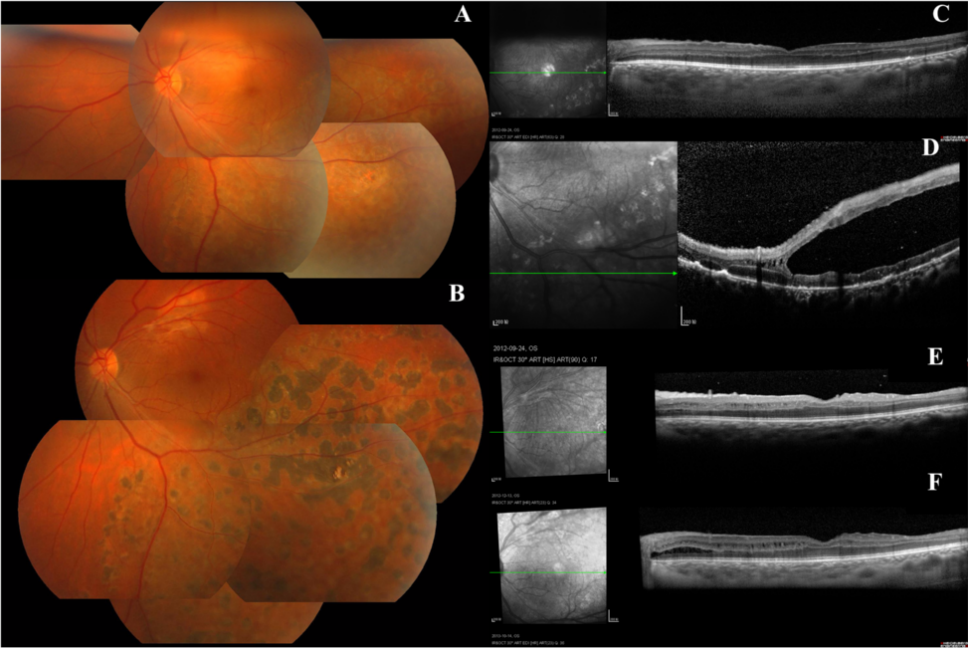
**The fundus photography and OCT follow up post-operation. A**: one-month after the bullous collapsed after surgery, the schisis cavity still remained in inferotemparal retina. **B**: FP OS: one-year follow up. the bulla collapsed, laser spot in schisis region. **C**: OCT OS: one-month follow up: macular region was flat. **D**: OCT OS: one-month follow up: schisis cavity was in inner nuclear layer in inferotemperal retina. **E**: OCT OS: three-month follow up: macular region was flat, schisis-like changes was at inner nuclear layer of retina. **F**: OCT OS one-year follow up: macular region was flat, schisis-like changes was at inner and outer nuclear layer of retina.

During follow up, the macular edema and folds disappeared. The BCVA was restored to 0.5 one month after surgery. BCVA stayed at 0.5 at 3 months after surgery, and increased to 0.6 at one year after surgery. The bulla collapsed and macular region was flat after surgery (Figure 2C) but the retinoschisis cavity in inferotemporal retina still remained (Figure 2A, B). The OCT at one month after surgery showed that the retinoschisis was in the inner nuclear layer (Figure 2D). OCT scans showed some schisis-like changes at inner and outer nuclear layers during the follow up (Figure 2E, F). Retinoschisis in right eye remained stable during follow up.

## Conclusion

Foveoschisis in nanothalmols or in PM has been reported. While previous reports present foveoschisis that was always combined with RPE abnormalities, or papillomacular folds caused by microphthalmos [1,4], in our case there were no signs of RPE abnormalities, or foveoschisis. Similar retinal findings have been described in association with nanophthalmos by Dhrami-Gavazi et al. in 2009 [7]. However, in his three cases, the schisis was just localized in peripheral. Paplliomacular folds are common in posterior nanophthalmos because of scleral growth independent of neuroretinal growth [1-3]. In our case, macular folds in left eye resulted due to cmprssion by bullous-like schisis. The macular was normal in the fellow eye.

Uveal effusion syndrome (UEF) and glaucoma have been reported to be very common in nanophthalmos. There were no symptoms of UEF in our case, such as cililary body detachment, leopard-spot in FFA, and retinal detachment. The eyes with nanophthalmos have thick sclera, uvea, and retina which may contribute to the irregular outflow of the eye, which causes the cystic dengeneration of the retina.

Although there have been some reports about the MFRP gene mutation correlated with the nanophthalmos [6,8], in our case, there was no relevant family history.

The management of our case was very challenging. According to our long-term observation of X-linked retinoschisis, in 82% (9 out of 11 eyes) of the eyes the schisis or retinal detachment progressed and visual acuity decreased during a mean follow-up of 34.7 months [9]. In vitrectomy group (n = 17 eyes), foveoschisis resolved in all eyes with resolution of retinal detachment in 94% (16 out of 17 eyes) of the eyes with recovery of visual acuity. Vitrectomy may be an effective and essential treatment for patients with progressive X-linked retinoschisis to prevent deterioration of vision. Though the mechanism is different between X-linked retinoschisis and acquired retinoschisis, the schisis in our case extended to posterior retina and visual acuity decreased. The outcome after surgery proved that vitrectomy is effective for preventing the schisis progression and deterioration of visual acuity.

In conclusion, in this rare case we found that if schisis is limited to the peripheral retina it may be stable but, if it progressed to the posterior pole, vitrectomy may prevent the progression of acquired retinoschisis.

## Consent

Written informed consent was obtained from the patient for publication of this Case report and any accompanying images. A copy of the written consent is available for review by the Editor of this journal.

## Abbreviations

PM: Posterior microphthalmos; OD: Right eye; OS: Left eye; OU: Both eyes; BCVA: Best correct visual acuity; FFA: Fluorescence angiography; UEF: Uveal effusion syndrome; OCT: Optical coherence tomography.

## Competing interests

All authors declare that they have no financial or non-financial competing interests.

## Authors’ contributions

The work presented here was carried out in collaboration between all authors. SSY was the major contributors in writing the manuscript. YG and YSH were the operator. XLL was the academic advisor. All authors read and approved the final manuscript.

## Pre-publication history

The pre-publication history for this paper can be accessed here:

http://www.biomedcentral.com/1471-2415/14/65/prepub
